# Y. M. D’s Soliloquy

**Published:** 1886-01

**Authors:** 


### Y. M. D's Soliloquy.

I would write for Daniel's red Journal,
Such papers as n'er were seen,
If it wouldn't expose how infernal
Incompetent I am—and how green;—
My pieces I'd make scientific,
And prepare with elaborate pains,
They'd be read e'en to the Pacific,
But hang it! I haven't the brains.

—Dr. Merryman.

